# Report from a text-based blood pressure monitoring prospective cohort trial among postpartum women with hypertensive disorders of pregnancy

**DOI:** 10.1186/s12884-024-06511-1

**Published:** 2024-05-03

**Authors:** Ahmed S.Z. Moustafa, Wondwosen Yimer, Ana Perry, Lucia Solis, Sheila Belk, Rachael Morris, Shauna-Kay Spencer, Sarosh Rana, Kedra Wallace

**Affiliations:** 1 Department of Obstetrics & Gynecology, University of Mississippi Medical Center, Jackson, MS USA; 2 Department of Data Science, University of Mississippi Medical Center, Jackson, MS USA; 3 Department of Medicine, University of Mississippi Medical Center, Jackson, MS USA; 4 Department of Pharmacology &Toxicology, University of Mississippi Medical Center, Jackson, MS USA; 5 Department of Obstetrics & Gynecology, University of Chicago, Chicago, IL USA; 6 Myrlie Evers Williams Institute for the Elimination of Health Disparities, University of Mississippi Medical Center, Jackson, MS USA

**Keywords:** Blood pressure monitoring, Hypertensive disorder, Postpartum hypertension, Preeclampsia, Remote, Teletext

## Abstract

**Background:**

Hypertensive disorders of pregnancy are a main cause of maternal morbidity and mortality in the United States and worldwide, and it is estimated that approximately 60% of maternal deaths in the United States occur during the postpartum period. The utilization of telehealth modalities such as home blood pressure monitoring has shown improvement in blood pressure control and adherence with follow up visits. Our study sought to determine if standardized education improved patient hypertension knowledge and if this when combined with home blood pressure telemonitoring increased participants’ postpartum self-blood pressure monitoring and postpartum visit attendance.

**Methods:**

This is an Institutional Review Board approved prospective cohort study conducted at the University of Mississippi Medical Center. Women with a hypertensive disorder of pregnancy who met the inclusion criteria and provided written informed consent to participate were enrolled. Participants received a baseline pre-education questionnaire designed to assess their knowledge of their hypertensive diagnosis, hypertension management, and postpartum preeclampsia (PreE). Participants then received standard education, a blood pressure monitor, and were scheduled a follow-up visit during the first 10 days following discharge. Remote home blood pressure monitoring was performed via text messages and voice calls for 6-weeks postpartum. At the conclusion of the study, participants repeated their original questionnaire.

**Results:**

250 women provided informed consent to participate in the study and were included in this analysis. Relative to the baseline survey, there was a significant increase (*p* = 0.0001) in the percentage of correct responses. There was not an association between study engagement and percentage of correct responses on end of study questionnaire (*p* = 0.33) or postpartum visit attendance (*p* = 0.69). Maternal age was found to drive study engagement, even when adjusted for community-level distress (*p* = 0.03) and maternal race (*p* = 0.0002).

**Conclusion:**

Implementing a standardized postpartum education session was associated with improvement in patient’s knowledge. Further studies are needed with more longitudinal follow up to assess if this program would also result in improved long-term outcomes and decreased hospital readmission rates.

**Trial registration:**

NCT04570124.

**Supplementary Information:**

The online version contains supplementary material available at 10.1186/s12884-024-06511-1.

## Introduction

Pregnancy-related mortality rates in the United States (US) have increased each year since the Pregnancy Mortality Surveillance System was implemented in 1987 [1]. Hypertensive disorders of pregnancy (HDP) complicate 5–10% of pregnancies [2] and are a major cause of maternal morbidity and mortality in the US and worldwide [3]. It is crucial to recognize that approximately 60% of maternal deaths in the US occur in the postpartum period [3, 4], with up to 65.8%of these deaths believed to be preventable [3, 4]. In this report, a maternal death was considered to be preventable if there was at least some chance of avoiding such outcome by a change to patient, community, provider, facility, and/or system factors [4, 5]. These studies and others have led to an increased awareness of the importance of the postpartum period.

Recognizing the significant risk of morbidity in the early postpartum period especially among pregnancies complicated by HDP, the American College of Obstetricians and Gynecologists (ACOG) recommends early postpartum blood pressure evaluation [5]. This further complicates management of women diagnosed with a HDP as they need more frequent visits and heightened surveillance in the postpartum period. A secondary analysis of 115,502 women in 25 US hospitals over 3 years attributed hypertensive complications to 20.5% of cases of severe maternal morbidity [6]. Despite the efforts to mitigate the rising rates of maternal mortality in the US, there are still no standardized guidelines specifically addressing the optimal management of pregnancies complicated by HDP in the postpartum period. This leaves a large percentage of women vulnerable at a critical time, while also leaving healthcare providers with no clear guidance regarding their medical management.

In another report from nine maternal mortality review committees, provider factors and patient factors accounted for 51.8% and 23.2%, respectively, of the total contributing factors for preeclampsia and eclampsia related deaths [7]. Almost 70% of these factors were related to communication and knowledge leading the committees to recommend an improvement in patient and provider communication and coordination as well as improving standards related to assessment, diagnosis and policies related to prevention initiatives [7]. Health literacy (the degree to which individuals have the capacity to obtain, process, and understand the basic health information and services they need to make appropriate health decisions” [8]) has been shown to correlate with health outcomes [9]. Importantly, limited health literacy is ubiquitous, and a growing body of literature demonstrates a correlation between health literacy and health outcomes 1. Adults with low health literacy are at increased risk of hospitalization, encounter barriers to accessing health services, and are less likely to understand medical advice. ACOG describes the roles of the obstetrician in addressing health literacy such as tailoring health information to intended users and developing written material among other roles [9]. It is also suggested that the use of remote blood pressure monitoring (BPM) and surveillance with text messaging may improve adherence for BPM in the postpartum period [10]. Aiming to address some of these issues, our collaborators at the University of Chicago developed the Systematic Treatment And Management of PostPartum Hypertension (STAMPP HTN) quality improvement initiative [11]. In this initiative, problems in the care of women with HDP were identified specifically at the time of delivery admission and postpartum and solutions were proposed. The STAMPP HTN bundle included education for healthcare professionals, patient education, dedicated nurse educator for postpartum appointments scheduling, protocols for management of patients with HDP in the inpatient, outpatient, and readmission setting [11]. In this study, the authors reported that implementing the STAMPP HTN bundle was associated with improved blood pressure control in the postpartum period, and higher attendance of postpartum visits (PPV). Incorporation of telehealth led to further improvement of rates of postpartum hypertension follow-up and elimination of disparity [12].

Some studies have shown that home BPM is well accepted among patients and is associated with better compliance with follow up visits [13–15]. Other studies have shown that using standard tools for patient’s education is associated with improved knowledge [16, 17]. Women with adverse social determinants of health, or higher levels of community distress (i.e. unemployment and job insecurity, housing insecurity, less access to resources) are often at higher risks for HDP [18, 19]. Therefore, the objective of this current study was to determine if implementing a program that focuses on patient blood pressure education following a HDP will improve patient’s blood pressure knowledge and adherence to BPM, and if community distress was a factor. Highlighting the importance of the intersection between knowledge and communication in the postpartum period, we sought to implement a standardized approach of management that focuses on education, improving patient’s knowledge, and communication with providers. We adapted the STAMPP-HTN program that was developed by our collaborators at the University of Chicago [11, 12] to our population of women. We hypothesize that implementing the STAMPP-HTN program for management of pregnancies complicated by HDP in the postpartum period that focuses on patient education and incorporates remote BPM will improve patient hypertension knowledge and PPV attendance.

## Methods

### Study design

This is an Institutional Review Board approved (IRB 2020 − 0175; NCT04570124, trial registration 30/09/2020) prospective study conducted at a single center, the Winifred Wiser Hospital (Women’s Hospital) at the University of Mississippi Medical Center. Patients seeking care at the the Wiser Hospital are predominantly black (65.1%) or white (22%) with a small percentage of Hispanic women (0.34%). This hospital services a high percentage of Medicaid recipients, 53.28% with only 26.91% of women having private insurance and the remaining either being self-pay or no pay.

Participants were recruited and enrolled beginning 14/12/2020 through 28/06/2021. Written informed consent was obtained from all study participants who met the inclusion/exclusion criteria. Women between the ages of 18–45 years of age were eligible if they were diagnosed with a HDP (the following diagnosis as defined by ACOG were eligible for a HDP: gestational hypertension, preeclampsia, chronic hypertension, HELLP syndrome, and women with sustained elevated blood pressure) during their delivery hospitalization. Women also needed to be able to understand English or Spanish, be willing to use the blood pressure monitor and have access to the internet or phone service. Women were not included if they did not meet the inclusion criteria, had postpartum or postoperative complications that prolonged their hospital stay beyond postpartum day 10 or women who were participating in another clinical trial to evaluate blood pressure control.

### Study workflow

**Fig. 1:**
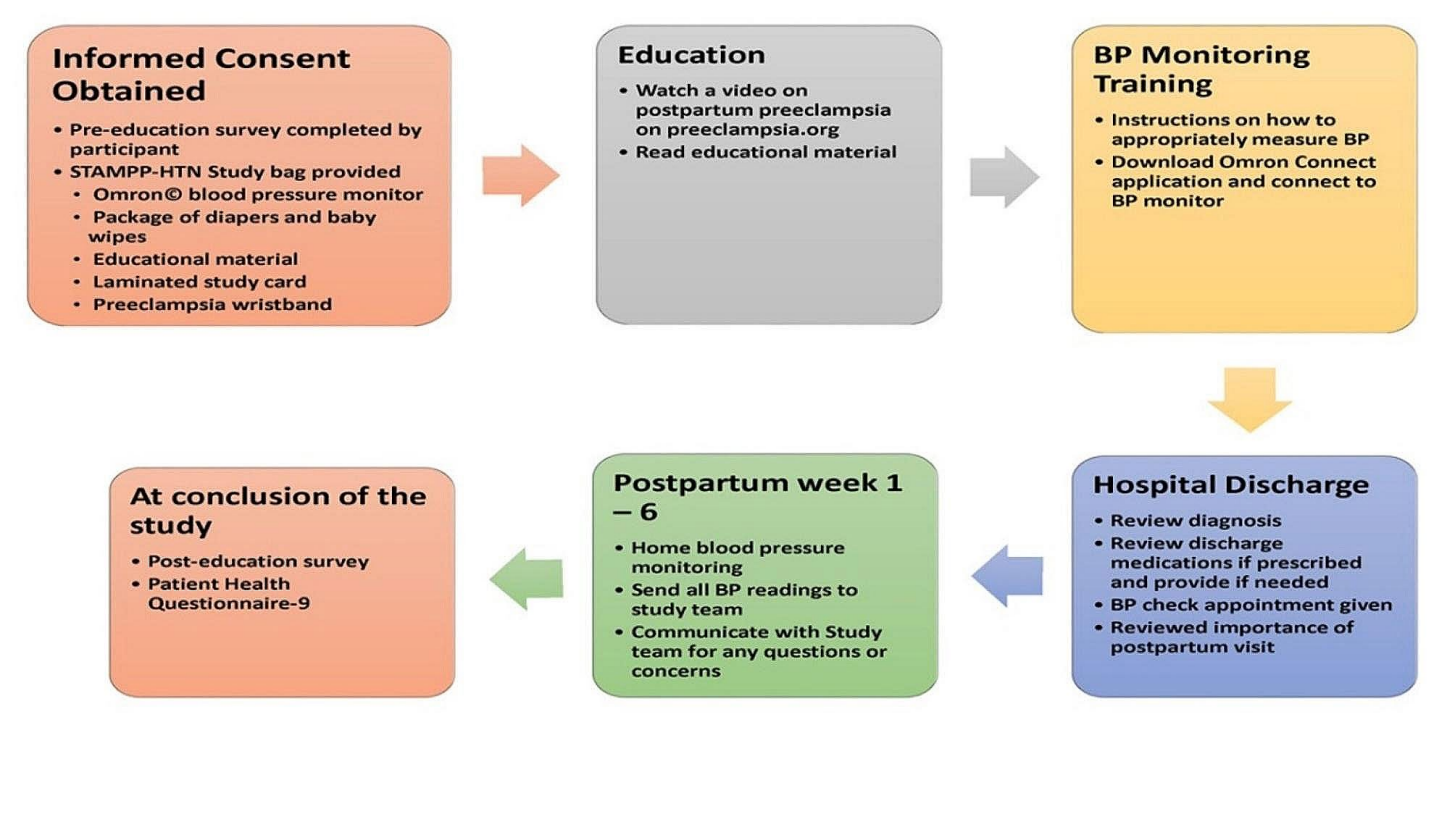
Flow diagram of study events

Following informed consent women received a pre-education survey designed to assess their knowledge of their hypertensive diagnosis, hypertension management, and postpartum preeclampsia and based on patient education material provided by the Preeclampsia Foundation (Fig. 1). All pre-discharge education and conversations were held in each participant’s private postpartum hospital room, where they were either alone or with a family member. As part of patient education women watched a 4-minute video on postpartum preeclampsia from the Preeclampsia Foundation (freely available on the Preeclampsia Foundation website (Preeclampsia.org), followed by a one-on-one review of their hypertensive diagnosis, which included coverage of material from the questionnaire. Questionnaires were designed by the study team to query the standard information shared with hypertensive patients both during pregnancy and in the post-partum period. A study team member then went over the educational material provided (also from the Preeclampsia Foundation) which highlights blood pressure goals, effects of hypertension after delivery and long-term adverse outcomes, the importance of postpartum follow up appointments, medication use, warning signs of postpartum preeclampsia, and instructions to call their provider if they develop any danger signs. Important to our study is the timing of consent and study procedures. For women who were admitted to the hospital for a scheduled induction or cesarean section, they were approached for consent prior to delivery and started the questionnaire and education portion of the study no earlier than 24 h postpartum. As a large number of women in the study required magnesium sulfate administration, women not already enrolled in the study were not initially approached until at least 36 h postpartum to allow everyone the chance to start the recovery from labor and delivery.

Next training on how to use the blood pressure machine and the Omron© Connect app was received. All instructions were also provided on the laminated study card. Prior to hospital discharge women received a STAMPP-HTN (labeled bag that included a blood pressure monitor (Omron©- wireless blood pressure monitor, HEM-9200T), and educational pamphlets on their HDP diagnosis (Available from the Preeclampsia Foundation). On their laminated study card information on their final diagnosis, antihypertensive medication regimen (if applicable), and their standard of care follow-up appointment for an in-person blood pressure check within 7–10 days postpartum was provided. Important to this study, is the fact that even though this is a standard of care follow-up appointment, it was noted that this visit was not routinely scheduled for all hypertensive patients at our hospital. As such, all women in this study had this visit scheduled. The card also contained emergency phone numbers (inclusive of study team), and participants received a study identification STAMPP-HTN wristband. In coordination with our hospital’s pharmacy, we provided antihypertensive medications free of charge for study participants who were prescribed antihypertensive medications on discharge and indicated to their clinical care team a financial barrier in filling their prescription.

### Remote BPM

Following discharge, women were asked to measure their blood pressure and send their readings to the study team via text message following the timeline outlined in Table 1.

**Table 1:** Frequency of remote blood pressure reading submissions

Postpartum Week 1	Submit blood pressure 1x AM and PM for 7 consecutive days
Postpartum Weeks 2–6	Submit blood pressure 1x AM and PM 1 day a week

All blood pressure readings were reviewed in real-time, 24 h a day and 7 days a week, by the study team as they were received. For women with readings ≥ 150mmHg systolic and/or ≥ 100mmHg diastolic they were asked to retake their pressures and questions were asked regarding adherence to medication, activities prior to blood pressure reading, etc. If their follow-up blood pressure was within normal ranges, they were advised to check their blood pressure again later that day and to contact the study team if the subsequent reading was also high. In the event the follow-up blood pressure was also high, study team physicians were made aware of any consecutive elevated blood pressure readings. These participants received a phone call by the study physician to discuss further management. Any management plans necessitating hospital presentation or medication adjustment was conveyed to the participants’ physician.

Once discharged, all communication took place via text, unless it was a physician call, the participant requested a member of the study team to call them or the study team was receiving/returning a phone call.

### Post-BPM procedures

At the end of the 6-week BPM, participants received a post-education survey that had the same questions as the pre-education survey. All surveys were administered in person, by telephone or sent via mail or email. For study participation all women were allowed to keep their blood pressure machines and received compensation for their time as follows: Baseline questionnaire and blood pressure training (Pamper diapers and baby wipes), completion of week 1 blood pressure monitoring ($25 gift card), completion of blood pressure monitoring for weeks 2–5 ($5 gift card per completed week), completion of week 6 blood pressure monitoring and end of study surveys ($15 gift card).

### Post-study roll off

At the time of study consent, each participant listed their primary care physician and consented to their physician receiving a letter detailing the study, their final hypertensive diagnosis, their hypertensive medication at discharge, any notated changes made in dosage during the study, their blood pressure at discharge and their final study blood pressure. Due to the rural population of our state and the lack of electronic medical records at all facilities, this information was sent to help encourage communication between primary care physicians and the study participant regarding blood pressure management. The study participant also received this same information at the end of the study, regardless of their study participation.

### Data entry and collection

All data was collected from participants’ electronic medical records, and was managed using REDCap (Research Electronic Data Capture) electronic data capture tools [20, 21].

### Data analysis

Summary statistics are presented as mean ± standard deviation for continuous variables and frequencies, interquartile range (IQR) and percentages for categorical variables. Chi-squared test, Fisher’s exact, Kruskal-Wallis, and Wilcoxon Two-Sample test were used for the summary analysis. Analysis of Variance was used to compare the mean of different groups. Univariable and multivariable linear or logistic regression was used, as appropriate, to evaluate performance on the survey as well as responses in blood pressure monitoring. In anticipation of decreased study participation by week 6, we over recruited women to ensure 60 women, number needed for statistical power, remained in the study at 6 weeks. Statistical software SAS version 9.4 (SAS Institute INC., Cary, NC) and GraphPad Prism (Boston, MA) were used to perform analyses. A two-sided *P* < 0.05 was used to indicate statistically significant results.

## Results

**Fig. 2:**
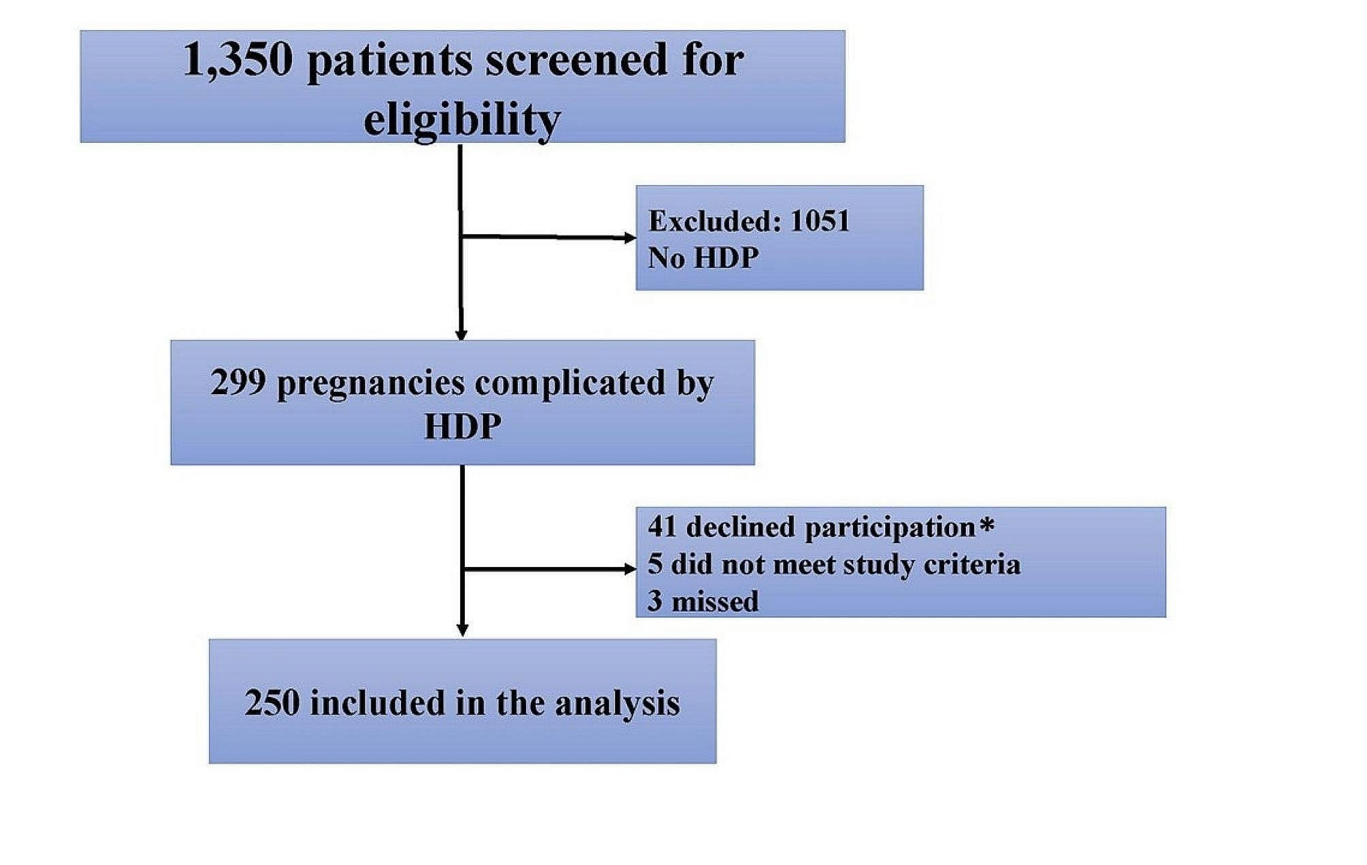
Flow diagram of patients screened and enrolled into the study. *Most common reasons given for declining participation were: no interest in research and did not have the time to complete study procedures

Of the 1,350 women screened for this study, a total of 250 women were enrolled (Fig. 2). 73.6% of study participants were Black, 79.8% classified as Class I obese or greater, and 61.5% were Medicaid recipients. The median (IQR length of maternal hospital stay was 2.88 days (2–3 days). The average birthweight was 2,518.26 ± 875.65 g with 36.64% (*n* = 96) of infants admitted to the neonatal intensive care unit.

During the study period, 22 women were readmitted to the hospital, with 77.2% (*n* = 17) being admitted for postpartum hypertensive disorders (Table 2). 2 women were admitted for abscess, 1 for a non-hypertensive headache, 1 with peripartum cardiomyopathy and 1 participant for COVID pneumonia.

**Table 2:** Readmission data for the 17 women admitted for hypertensive crises. Data is expressed as mean (range) or as a range

Diagnosis	Postpartum day	Peak systolic range	Peak diastolic range
Hypertension			
PE or cHTN (*n* = 2) PE with SF (*n* = 6) SI-PE with SF (*n* = 9)	21.5 (6–37) 16 (7–45) 13.44 (7–13)	169–214 141–199 162–211	107–120 91–116 85–113

cHTN – chronic hypertension; PE – preeclampsia; SF – severe features

As there is a relationship between health literacy and access to care, we evaluated whether study participants resided in areas notated for having low resources (i.e. distressed communities [22]). The majority of the study population (77%, *n* = 193) live in at-risk or distressed communities, compared to 17% (*n* = 43) who lived in prosperous to comfortable communities. Additional details regarding the baseline characteristics of women enrolled in this study can be found in Table 3.

**Table 3:** Characteristics of Participants

Characteristics	Participants
**Maternal age (yrs)**	28.16 ± 6.1
**Race**	
Black	193 (73.66%)
White	45 (17.18%)
Hispanic	7 (2.67%)
Other	5 (1.91%)
**BMI**	
x ≤ 24.9 kg/m^2^	8 (3.05%)
25.0–29.9 kg/m^2^	33 (12.60%)
30.0 ≥ x kg/m^2^	209 (83.6%)
**Insurance**	
Medicaid	161 (64.40%)
Private	76 (30.4%)
None/Self-pay	1 (0.40%)
Unknown	12 (4.80%)
**Parity**	
Nulliparous	90 (36.0%)
Multiparous	160 (64.0%)
**Gestational Age at Delivery (wks)**	35.66 ± 3.81
**Preterm Delivery (< 37 weeks)**	113 (45.2%)
**Mode of Delivery**	
Cesarean delivery	151 (60.40%)
Vaginal delivery	99 (39.60%)
**Distressed Community Index**	
Prosperous	11 (4.4%)
Comfortable	32 (12.8%)
Mid-tier	14 (5.6%)
At risk	35 (14%)
Distressed	158 (63.2%)

Data presented as n (%), mean ± SD

250 participants completed the pre-education baseline survey, with on average 25.5 ± 15.9% of questions being answered correctly (Table 4). 23.6% (*n* = 59) of participants did not respond to any text messages, phone calls or mail outs during the study period leaving 191 women actively engaged in the study. Weekly study engagement is reported in the data supplement as is initial survey responses based on hypertensive diagnosis. From these women 48.7% (*n* = 93) completed the post-study survey. Compared to their initial responses (26.21 ± 14.87% correct *n* = 93), women had a significant increase in the percent of correct responses on the post-study survey (34.95 ± 17.34% correct; *p* < 0.0001, *n* = 93).

**Table 4:** Survey questions administered to study participants. The correct percentage of Baseline are indicated

Questions	Baseline
My doctor told me I had ? (choose a diagnosis) a. Gestational hypertension b. Preeclampsia c. Preeclampsia with severe features d. Superimposed preeclampsia e. Chronic hypertension f. Superimposed preeclampsia with severe features g. HELLP Syndrome h. None of the above	24.2%
High blood pressure or hypertension will go away now that I had my baby? (true/false)	33.9%
Having headaches that do not get better with Tylenol and spots in my eyes are normal after having a baby? (true/false)	15.7%
If I am checking my blood pressure I should repeat it if my top number is 150 or more and my bottom number is 100 or more? (true/false)	10.7%
If I did not have preeclampsia during pregnancy, I cannot get preeclampsia after delivery (true/false)	23.8%
If I feel good and can do the things I did before I had my baby I do not need to go to my postpartum visit (true/false)	6.8%
When can I stop taking my blood pressure medications? a. When my blood pressure is good for 4 weeks in a row b. If it makes me sick c. When my doctor says it is okay d. Never	70.3%
My blood pressure problems in pregnancy increases my cardiovascular lifetime risks? (true/false)	28.6%

### Impact of study engagement on learning

We evaluated participants’ engagement with the BPM program by assessing the number of day’s blood pressure readings were submitted to the study team throughout the 6-weeks of the study. The required number of days for a 100% response rate (i.e. study engagement) was 12 days (daily for the first week, then once a week for the remaining 5-weeks). The median number of days participants responded was 7 (IQR: 4–11).

**Fig. 3:**
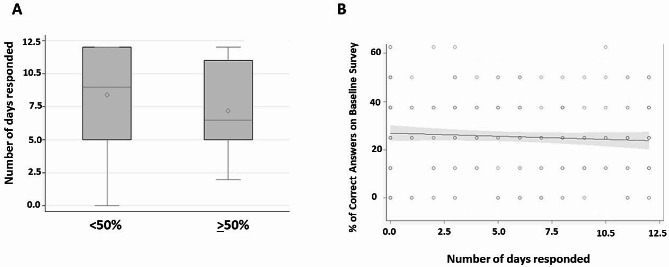
Study engagement and survey performance. Post-study survey scores were ranked as < 50% or ≥ 50% correct and compared to the number of days blood pressure reading was submitted. The diamond symbols and the horizontal lines in the box interiors represent the group means and the group medians, respectively. (**A**). The association between the percent of correct answers on the baseline survey and the number of times women submitted a blood pressure reading. The shaded areas around the green best fit line represents the 95% confidence interval (**B**)

To determine if there was a relationship between engagement in the study and test scores, we grouped women into those who scored < 50% or ≥ 50% correct answers on their end of study survey. We tested the association between the number of days participated in the study and the percent of correct responses on the end of study survey. Women who were more engaged were not more likely to answer more questions correctly (*p* = 0.33) compared to women who answered fewer questions correctly (Fig. 3A). There was not a significant association between the percentage of questions answered correctly on the baseline survey and study engagement (*p* = 0.26, Fig. 3B).

### Impact of sociodemographics on communication, learning and study participation

Less than 50% (*n* = 104) of women were able to correctly recall the type of hypertensive pregnancy condition they had (Question #1) when queried during the immediate post-partum period. When we specifically looked into this question there was no statistically significant differences in the percentage of correct responses when we compared women who live in prosperous, comfortable, and mid-tier communities (47.3%) compared to women who live in at-risk, and distressed communities (42.9%, *p* = 0.56). We next evaluated the performance on the baseline survey by community-level distress. On average, the percentage of correct answers was higher among women living in more distressed communities by 4.6% compared to women living more prosperous communities (*p* = 0.06). Similarly, among women who answered the post-study survey questionnaire there was not an association between the percentage of correct answers and their level of community distress (*r* = 0.05, *p* = 0.61).

Women living in prosperous to mid-tier communities were significantly more likely engage in the study relative to women living in more distressed communities (*p* = 0.001). When we controlled for community-level distress we found that Black or Hispanic women were more likely to have less participation (*p* < 0.0001) relative to non-Black or non-Hispanic women at the same level of community distress, indicating that maternal race and ethnicity also contributes to study participation. Among women who answered the post-survey questionnaire there was not an association between the percentage of correct answers and maternal race (*r* = 0.002, *p* = 0.99).

When we examined the relationship between response and age we saw that women who were younger were less likely to engage in the study (*p* = 0.03). When community-level distress was controlled this relationship remained true for women living in a less distressed community (*p* = 0.03) and for those who were not Black or Hispanic (*p* = 0.0002). However, when these same factors controlled for age, there was no longer a significant response (*p* = 0.13) indicating that maternal age is driving the study participation. Interestingly, among women who answered the post-survey questionnaire there was a negative association between the percentage of correct answers and maternal age, suggesting that younger women were more likely to have more correct answers on the post-survey questionnaire (*r*=-0.23, *p* = 0.02).

### Impact of study participation on PPV attendance

Overall 39.2% (*n* = 98) of enrolled study participants attended the in clinic postpartum blood pressure check that was scheduled visit within their first 10 days postpartum. Prior to this study, the scheduling of this visit was provider dependent and not based on maternal diagnosis. Among the 79 patients whose scheduled 6-week PPV at our facility was reported, 61 (77%) attended their standard of care PPV (Table 5), which aligns with the 75–78% typically reported for this population of women.

**Table 5:** 6-week postpartum attendance visit for our study population

Status	Number (%)
Attended visit	61 (24.4)
Did not attend scheduled visit	18 (7.2)
Scheduled at an outside clinic and attendance is unverified	118 (47.2)
No appointment listed in EHR	53 (21.2)

EHR – electronic health record

There was not a statistically significant difference when we examined the relationship between study participation and PPV attendance (*p* = 0.69). Women who did not attend their PPV participated in the study 6.5 days [IQR 0–9] vs. 7 days [IQR 1–11] for women who did attend their PPV. We also evaluated if performance on the baseline survey affected rates of PPV attendance. We compared PPV attendance between women who answered ≥ 50% of questions correctly (88.9% attended) to those who answered < 50% correctly (78% attended) and saw no difference between the groups (*p* = 0.67).

When we examined sociodemographic factors we found no relationship between community-level of distress and the 6-week standard of care PPV attendance. Among the 79 women with scheduled visits (Table 5), 20.5% of women not attending lived in communities of moderate to no distress (*n* = 7/34) compared to 24.4% of women living in communities with high levels of distress (*n* = 11/45) *p* = 0.69). We next determined if there was an association between PPV attendance and the percent of questions answered correctly on the baseline survey and found no relationship (*p* = 0.70), which did not change when community-level of distress (*p* = 0.11) or maternal race (*p* = 0.57) was controlled.

## Discussion

In our study population, women living in communities with less distress had a lower percentage of correct answers when compared to women living in communities with a higher level of community distress. Distress was assessed using the distressed community index (DCI), which uses data based on the Census Bureau’s Business Patterns and American Community Survey. The collected data covers seven metrics (high school diploma, housing vacancy rate, adults not working, poverty rate, median income ratio, change in employment and change in establishments) which is used to sort zip codes into prosperous, comfortable, mid-tier, at risk or distressed [22]. There did not appear to be a significant difference in the overall performance of participants on the pre-education baseline survey when adjusting for race, or the community-level of distress. Our results showed an improvement in participants’ performance on the post-education survey completed at the conclusion of the study suggesting that providing standardized education enhanced the knowledge of the study participants. The percentage of correct answers on the post-education survey was significantly higher than on the pre-education survey. For example, more women knew that high blood pressure could still continue after delivery. This is crucial as observation of blood pressure is key in helping with the control of hypertension and decreasing the risk of complications and hospital readmissions. In a randomized control study by You et al. in which a standardized educational tool about preeclampsia was developed and compared to a standard ACOG pamphlet with no additional information, the authors noted improved knowledge regarding preeclampsia when this newly developed educational tool was used [16]. In a more recent study, Goel et al. evaluated the effectiveness of interactive education tools for pregnancies complicated by HDP [17]. In that study, the education intervention included 2 graphic-based education tools used for patient education. Similar to our findings, Goel et al. showed a slight improvement in patient’s perceived comprehension and objective knowledge using this intervention [17].

PPV attendance in the United States is estimated to be only at approximately 60% [5]. Pregnancies complicated by HDP require additional monitoring; ACOG recommends that women with a HDP have a blood pressure evaluation between 72 h and 10 days based on the severity of the diagnosis [5]. Therefore, we were also interested in determining if implementing a teletext BPM program in an under-resourced state would help increase blood pressure monitoring and PPV attendance. In our study, we noted very low adherence to the well-being blood pressure evaluation visit scheduled within the first 10 days postpartum at 39.2% with a 6-week attendance at 77% among the 41% of women we were able to acquire records for. Our PPV attendance was somewhat similar to the results observed by Hauspurg et al. who reported a PPV attendance of 88% for women enrolled in a remote BPM program compared to 66% prior to implementation [13]. In contrast to the low in-person 10 day blood pressure evaluation, 85% (*n* = 213/250) of the enrolled women participated in blood pressure submission during the first week of the study. As we did not query participants regarding barriers to participation or PPV attendance, we can only postulate that socioeconomic barriers (i.e. transportation) that have been recognized in our pregnant population as well as the fact a number of women who deliver at our hospital are transfer patients who return to their personal obstetricians following delivery may also influence this number. The decline in study engagement from week 1 through week 6, despite the text messages suggest that different means of team engagement will need to be identified to help increase participant engagement.

This study has multiple strengths. This is study was completed in in an under-resourced area that used an innovative teletext option that allowed women to manage their health with minimal interruption during the postpartum period. Women were also provided health modalities such as Bluetooth blood pressure monitoring to help them keep personal reading logs on their phones. Our study used a more comprehensive patient education program that included a one on one patient education session conducted by trained physicians and research staff, which incorporated watching a video, and using different educational materials and tools to maximize patients’ knowledge. Another strength of our study is our patient population which is largely Black and lived in distressed communities highlighting the additional challenges faced by this population. This suggests the need for mobilizing more resources and creating intensive education programs on a community level to address the racial disparities in observed maternal and neonatal outcomes.

We also recognize that our study has limitations. One of the limitations was the difficulty to verify standard of care PPV attendance for a large number of our participants. With our institution serving as the only highest-level referral center for the state, many of our participants live hours away and go back to their home town and primary providers after they safely deliver. Despite participants signing release of information consents to obtain records of their PPVs, we still had difficulty obtaining these records and even though our hospital system utilizes electronic health records that is not true for all areas of the state. There are also social factors which could have impacted PPV attendance which ranged from transportation to care for children at the house, both of which were comments that were made by study participants. Along these same lines was the exclusion of women who are younger than 18 and older than 45 at the time of childbirth. As such, future studies will increase the age range of eligible women, this will especially be important as there is an increased risk for preeclampsia with advanced maternal age [23]. However, it should be noted that in our population women who were younger in age and lived in communities with less distress were less likely to participate in the study, but when they did, they performed better on the post-study survey. This suggests, methods other than remote HBM might be needed to engage this demographic of women. As historically postpartum preeclampsia most frequently presents within the first 7–10 days [24], daily BP’s were only monitored for the first 7 days postpartum with a clinical blood pressure visit scheduled for day 10. However, the true risk of postpartum preeclampsia and/or readmission is increased with the first 6 weeks postpartum; as such future studies will need to consider an extended period of daily monitoring, especially as most women re-admitted were after postpartum day 10. As all education occurred in the immediate postpartum period it is important to note that, the health of the women and/or baby sometimes prevented us from approaching eligible women and that sometimes women were too tired or busy to engage in the study procedures.

## Conclusions

In summary, implementing the STAMPP-HTN educational bundle was associated with improvement in patient’s knowledge. To some degree sociodemographic factors such as community level of distress are associated with women’s engagement in a remote home blood pressure monitoring program. Further studies are needed with more longitudinal follow up to assess if this program would also result in improved long-term outcomes and decreased hospital readmission rates. While we did not assess barriers to implementation and participation among study participants, it was noted via the electronic records system, EPIC, that some study participants who did not interact with our study team did make calls to their doctors regarding high blood pressure readings at home. Finally, this study played a crucial role in identifying areas of need and gaps in managing pregnancies complicated by HDP within our system and community. This creates multiple opportunities for quality improvement projects including but not limited to periodic education of health care professionals regarding risks of HDP, enhancing patient education in the antepartum period especially for women at risk of developing HDP, improving access to home BP monitors, and facilitating postpartum visit scheduling.

### Electronic supplementary material

Below is the link to the electronic supplementary material.

Supplementary Material 1

## Data Availability

The datasets generated and/or analyzed during the current study are not publicly available due to patient confidentiality but may be available in a deidentified format from the corresponding author on reasonable request.

## Abbreviations

ACOG: American College of Obstetrics and Gynecology
BPM: Blood Pressure Monitoring
HDP: Hypertensive Disorder of Pregnancy
IQR: Interquartile Range
PPV: Postpartum visit
US: United States
