# Supplementary material for: Report from a text-based blood pressure monitoring prospective cohort trial among postpartum women with hypertensive disorders of pregnancy
Source: BMC Pregnancy Childbirth. 2024 May 3;24:340. doi: 10.1186/s12884-024-06511-1 (PMC11067202; doi:10.1186/s12884-024-06511-1)
Supplement: Supplementary file 1 — Supplementary Material 1 [file 12884_2024_6511_MOESM1_ESM.docx]

**Data Supplement.**

**Table S1.** Participant engagement

| **Number of women submitting blood pressures (Week 1)** | 213 |
| --- | --- |
| **Number of women submitting blood pressures (Week 2)** | 91 |
| **Number of women submitting blood pressures (Week 3)** | 54 |
| **Number of women submitting blood pressures (Week 4)** | 57 |
| **Number of women submitting blood pressures (Week 5)** | 44 |
| **Number of women submitting blood pressures (Week 6)** | 53 |
| **Number of women asked to repeat blood pressure** | 71 |
| **Number of women recommended to report to ER** | 24 |

**Table S2**. Participant responses to baseline questionnaire.

| **Questions** | **gHTN** | **PE** | **PE w/SF** | **cHTN** | **siPE** | **siPE w/SF** | **HELLP** |
| --- | --- | --- | --- | --- | --- | --- | --- |
| **My doctor told me I had?^[[1]](#footnote-1)^ (choose a diagnosis)**  **a. Gestational hypertension**  **b. Preeclampsia**  **c. Preeclampsia with severe features**  **d. Superimposed preeclampsia**  **e. Chronic hypertension**  **f. Superimposed preeclampsia with severe features**  **g. HELLP Syndrome**  **h. None of the above** | 28 (45.9%)  4 (6.6%)  0 (0%)  0 (0%)  0 (0%)  0 (0%)  0 (0%)  29 (47.5%) | 1 (20%)  4 (80%)  0 (0%)  0 (0%)  0 (0%)  0 (0%)  0 (0%)  0 (0%) | 7 (13.5%)  35 (57.6%)  7 (13.5%)  0 (0%)  3 (5.8%)  0 (0%)  0 (0%)  5 (9.6%) | 17 (27.9%)  9 (14.8%)  0 (0%)  0 (0%)  16 (26.2%)  0 (0%)  0 (0%)  22 (31.1%) | 0 (0%)  0 (0%)  0 (0%)  0 (0%)  0 (0%)  0 (0%)  0 (0%)  (100%) | 8 (18.6%)  29 (46.6%)  5 (11.6%)  0 (0%)  8 (18.6%)  1 (2.3%)  0 (0%)  1 (2.3%) | 0 (0%)  2 (40%)  0 (0%)  1 (20%)  0 (0%)  0 (0%)  4 (40%)  0 (0%) |
| **Q3 High blood pressure or hypertension will go away now that I had my baby?**  **True**  **False** | 33 (53.2%)  29 (46.8%) | 3 (50%)  3 (50%) | 32 (62.7%)  19 (37.3%) | 46 (71.9%)  18 (28.1%) | 1 (100%)  0 (0%) | 36 (81.8%)  8 (18.2%) | 3 (60%)  2 (40%) |
| **Q4 Having headaches that do not get better with Tylenol and spots in my eyes are normal after having a baby?**  **True**  **False** | 54 (85.8%)  9 (14.2%) | 6 (100%)  0 (100%) | 42 (80.8%)  10 (19.2%) | 53 (84.1%)  10 (15.9%) | 1 (100%)  0 (0%) | 37 (82.2%)  8 (17.8%) | 5 (100%)  0 (0%) |
| **Q5 If I am checking my blood pressure I should repeat it if my top number is 150 or more and my bottom number is 100 or more?**  **True**  **False** | 7 (11.5%)  54 (88.5%) | 2 (33.3%)  4 (66.7%) | 5 (9.8%)  46 (90.2%) | 5 (7.8%)  59 (92.2%) | 0 (0%)  1 (100%) | 3 (6.7%)  42 (93.3%) | 3 (60%)  2 (40%) |
| **Q6 If I did not have preeclampsia during pregnancy, I cannot get preeclampsia after delivery.**  **True**  **False** | 43 (69.4%)  19 (30.6%) | 3 (50%)  3 (50%) | 42 (80.8%)  10 (19.2%) | 49 (76.6%)  15 (23.4%) | 1 (100%)  0 (0%) | 36 (80%)  9 (20%) | 5 (100%)  0 (0%) |
| **Q7 If I feel good and can do the things I did before I had my baby I do not need to go to my postpartum visit**  **True**  **False** | 59 (93.7%)  4 (6.3%) | 6 (100%)  0 (0%) | 47 (92.2%)  4 (7.8%) | 58 (90.6%)  6 (9.4%) | 1 (100%)  0 (0%) | 43 (95.6%)  2 (4.4%) | 5 (100%)  0 (0%) |
| **When can I stop taking my blood pressure medications?^[[2]](#footnote-2)^**  **a. When my blood pressure is good for 4 weeks in a row**  **b. If it makes me sick**  **c. When my doctor says it is okay**  **d. Never** | 1 (1.5%)  4 (6.0%)  51 (76.1%)  11 (16.4%) | 0 (0%)  0 (0%)  3 (50%)  3 (50%) | 6 (11.8%)    3 (5.9%)  43 (74.5%)  4 (7.8%) | 3 (4.7%)  5 (7.8%)  49 (65.6%)  14 (21.9%) | 0 (0%)  0 (0%)  1 (100%)  0 (0%) | 0 (0%)    0 (0%)  32 (65.3%)  17 (34.7%) | 0 (0%)  0 (0%)  5 (100%)  0 (0%) |
| **Q9 My blood pressure problems in pregnancy increases my cardiovascular lifetime risks?**  **True**  **False** | 21 (33.9%)  41 (66.1%) | 1 (16.7%)  5 (83.3%) | 14 (26.9%)  38 (73.1%) | 21 (32.8%)  43 (67.2%) | 0 (0%)  1 (100%) | 7 (17.1%)  34 (82.9%) | 2 (40%)  3 (60%) |

1. Multiple answers possible for this question. [↑](#footnote-ref-1)
2. Multiple answers possible for this question. [↑](#footnote-ref-2)
